# Determinants of glycemic and blood pressure control in type 2 diabetes patients: 606 outpatients diabetes cohort

**DOI:** 10.1186/1758-5996-7-S1-A51

**Published:** 2015-11-11

**Authors:** Sabrina Coelli, Ariana Aguiar Soares, Ana Marina Moreira, Camila Kümmel Duarte, Luiza Barboza de Souza, Themis Zelmanovitz, Sandra Pinho Silveiro

**Affiliations:** 1 Universidade Federal do Rio Grande do Sul (UFRGS), Porto Alegre, Brazil

## Background

Diabetes mellitus (DM) complications are related to hyperglycemia, hypertension, smoking and lipids.

## Objectives

The aim of this study was to evaluate the determinants of metabolic and blood pressure levels in type 2 diabetes.

## Materials and methods

606 type 2 diabetes patients, outpatient Endocrine clinic, tertiary hospital, consecutively included between 2012-2014. Medical history, complete examination and laboratory evaluation performed (HbA1c, lipids, glomerular filtration rate -eGFR- and urinary albumin excretion-UAE). Therapeutic targets defined according to American Diabetes Association: HbA1c <7% (<8% if comorbidities); blood pressure <140/90 mmHg, total cholesterol (TC) <200 mg/dL, HDL adjusted for gender and triglycerides (TG) <150 mg/dL. Approved by ethics committee (nº 140073); statistical analysis PASW 20.0.

## Results

**Figure 1:**
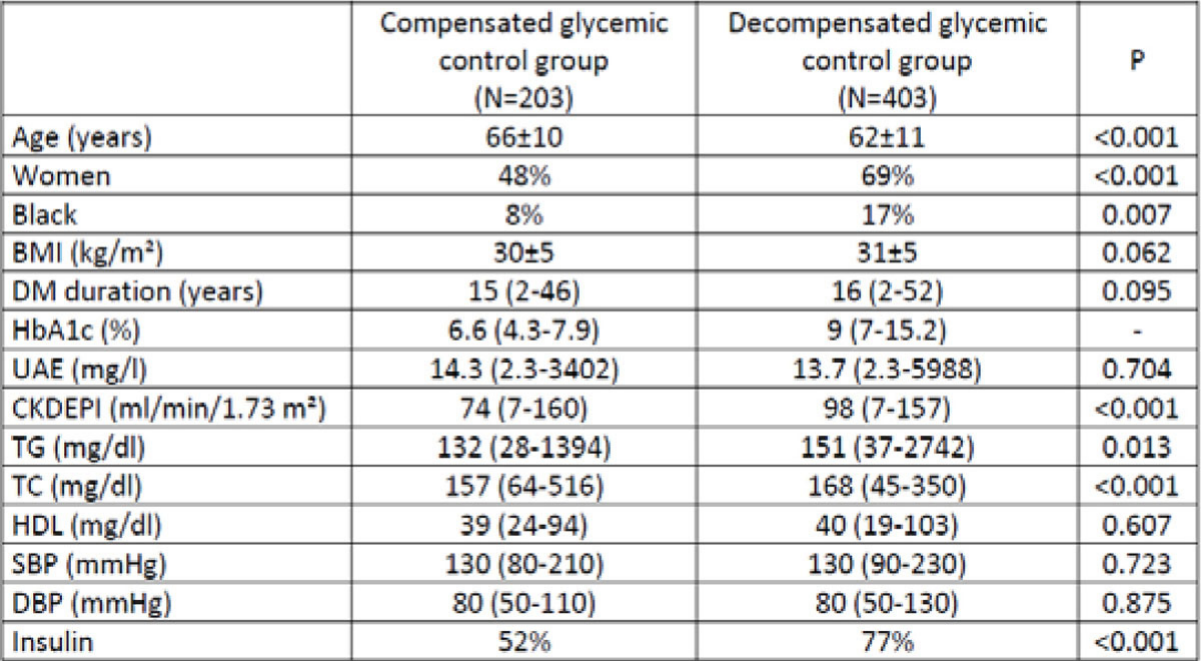
Comparisons between compensated and decompensated control groups.

The mean age was 63±11 yrs., 62% women, 86% white, 9% smokers, body mass index (BMI) 31±5 kg/m^2^, median DM duration 16 yrs. Median eGFR was 91 ml/min/1.73 m^2^, 50% with UAE >14 mg/l. Diabetes treatment: 2% diet only, 67% insulin (alone or in combination with oral agents). Regarding therapeutic targets: 54% and 78% presented systolic (SBP) and diastolic blood pressure (DBP), respectively, within the recommendations. Median HbA1c was 8% (4.3-15.2%), 36% were below the target. TC, TG and HDL were at the target in, respectively, 79%, 54% e 29% of the cases. The table shows the comparisons between compensated and decompensated glycemic control groups (Figure 1). Comparisons between gender did not show differences between age (P=0.61), DM duration (P=0.488) or blood pressure (P=0.117). However, women had higher BMI (P<0.001), worse lipid levels (TG: P=0.003; TC: P<0.001) and glycemic control (P<0.001).

## Conclusion

Two thirds of type 2 diabetes outpatients were above the recommended glycemic targets; obesity in women and black skin color were the main determinants for these findings.

